# Impact of comorbid borderline personality disorder on inpatient treatment for bulimia nervosa: analysis of routine data

**DOI:** 10.1186/s40479-018-0098-4

**Published:** 2019-01-16

**Authors:** Johannes Baltasar Hessler, Jörg Heuser, Sandra Schlegl, Tabea Bauman, Martin Greetfeld, Ulrich Voderholzer

**Affiliations:** 1 Schoen Clinic Roseneck, Am Roseneck 6, D-83209, Prien am Chiemsee, Germany; 2 Department of Psychiatry and Psychotherapy, University Hospital of Munich (LMU), Munich, Germany; 3 Department of Psychiatry and Psychotherapy, University Hospital of Freiburg, Freiburg im Breisgau, Germany

**Keywords:** Bulimia nervosa, Borderline personality disorder, Inpatient treatment, Eating disorder

## Abstract

**Background:**

A substantial rate of patients with bulimia nervosa (BN) also suffer from Borderline personality disorder (BN + BPD). It is widely unknown how these comorbid patients with BN + BPD present and respond to inpatient treatment. Aims of the study were to examine (1) specific characteristics of patients with BN + BPD at admission, discharge, and during treatment, and (2) differential effects of inpatient treatment for BN vs. BN + BPD.

**Method:**

We analyzed routine data of inpatients admitted for the treatment of BN between 2013 and 2017 in a specialized hospital for eating disorders. (1) Cross-sectional differences were examined with independent *t*-tests and χ^2^-tests**;** and (2) treatment effects pertaining to eating disorders symptoms, depression, psychosocial functioning and general psychopathology with repeated measures analysis of variance.

**Results:**

Of 1298 inpatients (96% female), 13.2% also had a diagnosis of BPD. (1) Patients with BN + BPD had more previous inpatient treatments (*p* = 0.001), had a longer length of stay (*p* = 0.003), gained more weight during treatment (*p* = 0.006), and were more often irregularly discharged (*p* = 0.018) as well as rated as unfit to work at discharge (*p* = 0.003). (2) Both groups improved in all examined variables (all main effects treatment *p* <  0.001). Patients with BN + BPD showed worse symptoms aggregated across admission and discharge (all main effects diagnosis *p* <  0.05). Patients with BN + BPD showed smaller improvements (interaction treatment×discharge) in depressive symptoms (*p* = 0.018), perfectionism (*p* = 0.009), and asceticism (*p* = 0.035) and discharge scores mostly lay in the range of the admission scores of the BN-only group.

**Conclusion:**

Patients with BN + BPD improve during intense and specialized inpatient treatment, yet, retain pronounced impairment at discharge despite longer treatment. Treatment needs to be improved and should focus on transdiagnostic symptoms of BN and BPD.

**Electronic supplementary material:**

The online version of this article (10.1186/s40479-018-0098-4) contains supplementary material, which is available to authorized users.

## Background

Bulimia nervosa (BN) is characterized by the consumption of large amounts of food in a short time period (binging), which is followed by attempts to remove the ingested food or reduce its caloric impact usually by vomiting, abusing laxatives and diuretics, as well as excessive exercising (purging). Even though patients usually retain normal weight, BN is a grave disorder with severe secondary symptoms, a tendency to chronification or migration to other disorders and a 10-year remission rate of only 50% under treatment [1].

Meta-analyses estimated one quarter [2] to one third [3, 4] of persons with BN to also meet criteria for Borderline Personality Disorder (BPD), which represented the highest comorbidity rate of all personality disorders in all eating disorders. Assuming a global prevalence of BN of 3.6 million [5], around 1 million persons would be expected to have both BN and BPD (BN + BPD). In turn, around eight % of a large sample with BPD also met criteria for BN, which was the second highest rate after eating disorder not otherwise specified [6].

The high comorbidity rate might not be surprising, as both BN and BPD potentially have interacting etiologies (e.g., BPD may promote BN, BN may exacerbate BPD) [7] and share impulsivity [8], affective instability [9, 10], as well as deficits in emotion regulation [11] as core symptoms. So far, only very little is known about the clinical presentation of persons with BN + BPD and their response to treatment.

Cross-sectional studies found that patients with BN + BPD compared to patients with BN had stronger general psychopathology and poorer functioning [12, 13] as well as more suicide attempts [14]. Yet, eating disorder symptoms were not found to be worse in the comorbid group [15].

Longitudinal studies are scarce. While a high rate of comorbid patients were remitted after 10 years, relapses and migrations to other eating disorders were common [16]. The treatment of BN was suggested to be severely complicated by a comorbid BPD diagnosis [13] and depressive symptoms [17] as well as general psychopathology [18] may lead to worse treatment outcomes in BN. Accordingly, two trials examined specific treatment programs for patients with both eating disorders and BPD. One trial examined the effect of Dialectical Behavior Therapy (DBT) adapted for eating disorders in patients who failed to respond to previous treatment [19]. While most patients improved, they showed low remission rates and high eating disorder pathology at the end of the trial and at a 15-month follow-up. The other trial investigated the outcome of mentalization-based treatment for comorbid patients [20]. While high drop-out rates complicated the interpretation of the results and more than ten % experienced an adverse event during the trial, those patients remaining in the study improved with regard to shape and weight concerns.

In sum, it is mostly unclear whether patients with BN + BPD present and respond differently to treatment than patients with BN alone [2]. Further knowledge of these aspects is crucial for improving treatment and outcomes for a relatively large group of patients that is potentially more impaired and has a worse prognosis than their non-comorbid counterparts. Following the findings presented above, our study aimed to investigate in a large sample of inpatients with BN (1) the specific characteristics of patients with comorbid BPD, and (2) differences in treatment course and outcome between comorbid and non-comorbid patients.

## Method

### Study sample

The sample consisted of patients that were admitted to the Schoen Clinic Roseneck in Prien, Germany, for the treatment of BN between January 01 2013 and December 31 2017. The hospital is highly specialized in the treatment of BN [21, 22] and other eating disorders. Diagnoses of BN and BPD according to ICD-10 were given by the treating therapists, who were experienced clinicians or therapists in training under supervision of experience clinicians.

Upon admission to the hospital, all patients signed informed consent to the use of their routine data for scientific purposes.

### Materials and procedure

All patients were administered a range of psychometric questionnaires both at admission and discharge. The Eating Disorder Inventory 2 (EDI-2 [23, 24]) assesses general eating disorder pathology and yields a total score and eleven subscores that cover typical eating disorder psychopathology. The subscores pertain to the following scales: drive for thinness, bulimia, body dissatisfaction, ineffectiveness, perfectionism, interpersonal distrust, interoceptive awareness, maturity fears, asceticism, impulse regulation, and social insecurity. The items are rated on a 6-point Likert-scale ranging from “always” to “never”.

Further, patients completed the Beck Depression Inventory-II (BDI-II; [25, 26]) and the Brief Symptom Inventory (BSI; [27, 28]). Among other measures, the BSI yields a Global Severity Index (GSI), which summarizes all information and gives a general estimate of the degree of psychopathology. All patients were rated on the Global Assessment of Functioning (GAF; described in the DSM-IV [29]), which describes a person’s level of psychosocial and occupational functioning with scores ranging from 1 to 100. Higher scores indicate better functioning.

### Inpatient treatment

The treatment for BN is described in more detail in [21]. All patients received an intense and multimodal inpatient treatment, which included CBT-based individual and group psychotherapy with disorder-specific modules (see, e.g., [30, 31]), as well as a range of additional groups. Individual and group psychotherapy were conducted by trained clinical psychologists and psychiatrists, who received regular supervision from experienced therapists.

Patients with statutory health insurance received one session and patients with private insurance two sessions of individual psychotherapy per week, each lasting 50 min. Individual psychotherapy was not manualized and exclusively disorder-specific, yet, always addressed psychoeducation on eating and eating disorders, individualized case formulation, changes in diet, body exposure and body acceptance, restructuring of cognitive biases, and relapse prevention.

Group treatment consisted of a manualized disorder-specific group, art therapy, sport therapy, social cooking, and social skills training. Patients with comorbid BPD also participated in a skills training group. Even though patients received meal time support (supervised eating), they were in charge of their own eating during the day and had always access to the hospital’s cafeteria, a supermarket, and the lavatories.

### Statistical analysis

Patients with BN and BN + BPD were compared on a range of variables pertaining to admission (age, weight in kg, number of previous inpatient and outpatient treatments, and length of the disorder in years), course of the treatment (length of stay in days, weekly and total weight gain in kg), and discharge (weight in kg) with *t*-tests for independent samples. Differences with regard to gender prevalence (female vs. male), the ability to work (fit for work vs. unfit for work vs. unclear) as assessed by the therapists, and the discharge reason (regular discharge vs. against medical advice vs. transfer to other hospital vs. other reasons) were compared with χ^2^-tests. Patients are regularly discharged when their symptoms have remitted to a degree that outpatient treatment can be justified. This procedure entails that patients still might show clinically significant symptoms at discharge. Transfers to other hospitals may result from any somatic or psychiatric indication, however, most often occur due to acute suicidality that requires a more protected setting. Other discharge reasons include “stress tests” in the patients’ usual environment to assess daily functioning, and death.

Differential treatment courses were examined with a range of 2 (within-factor treatment: admission vs. discharge) × 2 (between-factor diagnosis: BN vs. BN + BPD) repeated measures analyses of variance (RM-ANOVAs). The dependent variables were the EDI-2’s subscores and its total score, the BDI-II sumscore, the GSI, the GAF score, and BMI. Partial η^2^ is given as indicator of effect size, with values of 0.01 suggesting small, 0.06 medium, and 0.14 large effects. The degrees of freedom may vary between the different outcomes, as there were missing values for some instruments. Statistically significant interaction effects were followed-up with *t*-tests for dependent samples and respective Cohen’s *d* effect sizes were calculated.

## Results

Between 2013 and 2017, 1298 patients with BN were admitted for treatment in the Schoen Clinic Roseneck. Of these patients, 171 (13.2%) and had a comorbid diagnosis of BPD. Table 1 displays the group comparisons in the variables pertaining to admission, treatment course, and discharge. One patient in the BN group died during treatment. One-hundred sixty-nine patients had complete data for all outcomes.

**Table 1:** Characteristics of inpatients with BN and BN + BPD

Variable	BN	BN + BPD	*p*
Admission			
Age; *M* (*SD*)	25.41 (10.49)	26.03 (9.55)	0.466
Female gender; *N* (%)	1082 (96.0)	165 (96.5)	0.761
Weight (kg); *M* (*SD*)	62.21 (12.68)	63.61 (12.61)	0.282
Previous inpatient treatments; *M* (*SD*)	0.15 (0.36)	0.44 (0.50)	0.001
Previous outpatient treatments; *M* (*SD*)	0.74 (0.44)	0.73 (0.45)	0.944
Treatment course			
Length of stay (days); *M* (*SD*)	63.13 (33.47)	74.19 (45.48)	0.003
Weekly weight gain (kg); *M* (*SD*)	0.12 (0.40)	0.21 (0.37)	0.065
Total weight gain (kg); *M* (*SD*)	1.09 (3.60)	2.15 (3.80)	0.006
Discharge			
Weight (kg); *M* (*SD*)	63.75 (11.86)	65.99 (12.02)	0.077
Discharge reason; *N* (%)			0.018
Regular	940 (83.5)	131 (76.6)	
Against medical advice	24 (2.1)	2 (1.2)	
Transfer to other hospital	41 (3.6)	14 (8.2)	
Others	122 (10.8)	24 (14.0)	
Ability to work; *N* (%)			0.003
Fit for work	379 (33.7)	38 (22.2)	
Unfit for work	283 (25.1)	60 (35.1)	
Unclear	464 (41.2)	73 (42.7)	

*Note*. *BN* bulimia nervosa, BN + BPD bulimia nervosa with comorbid Borderline Personality Disorder, *p* statistical significance of the test statistics *t* for continuous variables and χ^2^ for categorical variables, *M* mean, *SD* standard deviation, *kg* kilograms

The results of the RM-ANOVAs suggest that both diagnostic groups improved with respect to eating disorder symptoms (Table 2), as well as depressive symptoms, general psychopathology, and psychosocial functioning (Table 3) from admission to discharge (main effect treatment). Also, both groups gained weight during treatment (Table 3). Aggregated across admission and discharge, patients with BN showed better eating disorder symptomatology, general psychopathology, depressive symptoms, as well as psychosocial functioning, and lower weight (main effect diagnosis). Only for the EDI-2 scores of interoceptive awareness and maturity fears there was no statistically significant difference between diagnoses. The slopes from admission to discharge were mostly similar for BN and BN + BPD (interaction effect diagnosis × treatment), except for the EDI-2 scores for perfectionism and asceticism, as well as depressive symptoms and BMI. Table 4 displays the decomposed interaction effects, which suggest that patients with BN and BN + BPD improved statistically significantly in all variables, yet, patients with BN + BPD showed smaller improvements and gained more weight (see mean differences and effect sizes). Additional file 1: Table S1 (Hessler_BN-BPD_SuppTable1.pdf) displays all descriptive statistics for the RM-ANOVAs.

**Table 2:** Results of the repeated measures analyses of variance for changes from admission to discharge in EDI-2 scores for inpatients with BN and BN + BPD

Variable	Effect	*F*,^1^ *p*	Partial η^2^
Total score	Treatment	385.56, < 0.001	0.31
	Diagnosis	76.51, < 0.001	0.08
	Treatment × Diagnosis	2.49, 0.115	< 0.01
Drive for thinness	Treatment	265.04, < 0.001	0.23
	Diagnosis	37.42, < 0.001	0.04
	Treatment × Diagnosis	2.83, 0.093	<
Bulimia	Treatment	665.90, < 0.001	0.43
	Diagnosis	6.70, 0.010	0.01
	Treatment × Diagnosis	0.02, 0.876	< 0.01
Body dissatisfaction	Treatment	114.26, < 0.001	0.12
	Diagnosis	34.46, < 0.001	0.04
	Treatment × Diagnosis	3.19, 0.074	< 0.01
Ineffectiveness	Treatment	205.52, < 0.001	0.19
	Diagnosis	83.80, < 0.001	0.09
	Treatment × Diagnosis	0.62, 0.433	< 0.01
Perfectionism	Treatment	49.22, < 0.001	0.05
	Diagnosis	7.08, 0.008	0.01
	Treatment × Diagnosis	6.90, 0.009	0.01
Interpersonal distrust	Treatment	111.37, < 0.001	0.11
	Diagnosis	41.14, < 0.001	0.05
	Treatment × Diagnosis	0.34, 0.558	< 0.01
Interoceptive awareness	Treatment	186.73, < 0.001	0.18
	Diagnosis	1.64, 0.201	< 0.01
	Treatment × Diagnosis	1.64, 0.201	< 0.01
Maturity fears	Treatment	50.78, < 0.001	0.06
	Diagnosis	3.31, 0.069	< 0.01
	Treatment × Diagnosis	0.29, 0.591	< 0.01
Asceticism	Treatment	131.04, < 0.001	0.13
	Diagnosis	25.21, < 0.001	0.03
	Treatment × Diagnosis	4.47, 0.035	0.01
Impulse regulation	Treatment	92.79, < 0.001	0.10
	Diagnosis	106.96, < 0.001	0.11
	Treatment × Diagnosis	0.09, 0.760	< 0.01
Social insecurity	Treatment	158.33, < 0.001	0.16
	Diagnosis	61.03, < 0.001	0.07
	Treatment × Diagnosis	0.86, 0.353	< 0.01

*Note*. ^1^degrees of freedom for all tests = 1, 874, except for asceticism (1, 865), impulse regulation (1, 866), and social insecurity (1, 865). *EDI-2* Eating Disorder Inventory 2, *BN* bulimia nervosa, *BN* + *BPD* bulimia nervosa with comorbid Borderline Personality Disorder

**Table 3:** Results of the repeated measures analyses of variance for changes from admission to discharge in BDI-II, GSI, GAF, and BMI for inpatients with BN and BN + BPD

Variable	Effect	*F* (*df*), *p*	Partial η^2^
BDI-II	Treatment	418.94 (1, 672), < 0.001	0.38
	Diagnosis	70.59 (1, 672), < 0.001	0.10
	Treatment × Diagnosis	5.60 (1, 672), 0.018	0.01
GSI	Treatment	174.67 (1, 984), < 0.001	0.15
	Diagnosis	74.28 (1, 984), < 0.001	0.07
	Treatment × Diagnosis	2.76 (1, 984), 0.097	< 0.01
GAF	Treatment	228.35 (1, 365), < 0.001	0.39
	Diagnosis	8.55 (1, 365), 0.004	0.02
	Treatment × Diagnosis	0.75 (1, 365), 0.387	< 0.01
BMI	Treatment	72.15 (1, 721), < 0.001	0.09
	Diagnosis	2.56 (1, 721), 0.110	< 0.01
	Treatment × Diagnosis	7.50 (1, 721), 0.006	0.01

*Note*. *df* degrees of freedom, *BDI-II* Beck Depression Inventory II, *GSI* Global Severity Index, *GAF* Global Assessment of Functioning, *BMI* body mass index, *BN* bulimia nervosa, *BN + BPD* bulimia nervosa with comorbid Borderline Personality disorder

**Table 4:** Decomposition of the statistically significant interaction effects from the repeated measures analyses of variance. Results of the *t*-tests for dependent samples

Variable	*M* (*SD*) admission − discharge		*t*(*df*), *p*	*Cohen’s* d
EDI2: perfectionism	BN	0.29 (0.57)	14.24 (774), < 0.001	0.42
	BN + BPD	0.13 (0.58)	2.28 (100), 0.024	0.17
EDI2: asceticism	BN	0.37 (0.52)	19.69 (766), < 0.001	0.73
	BN + BPD	0.25 (0.45)	5.61 (99), < 0.001	0.45
BDI-II	BN	14.70 (11.30)	31.53 (586), < 0.001	1.28
	BN + BPD	11.66 (10.59)	10.27 (86), < 0.001	0.98
Body mass index	BN	−0.40 (1.28)	−7.82 (622), < 0.001	−0.09
	BN + BPD	− 0.78 (1.37)	−5.71 (99), < 0.001	−0.18

*Note*. After Bonferroni-correction for multiple testing, the significance level for these tests is at 0.05/8 = 0.006. *M* Mean, *SD* standard deviation, *EDI-2* Eating Disorder Inventory 2, *BDI-II* Beck Depression Inventory II, *BN* bulimia nervosa, *BN + BPD* bulimia nervosa with comorbid Borderline Personality Disorder

**Fig. 1:**
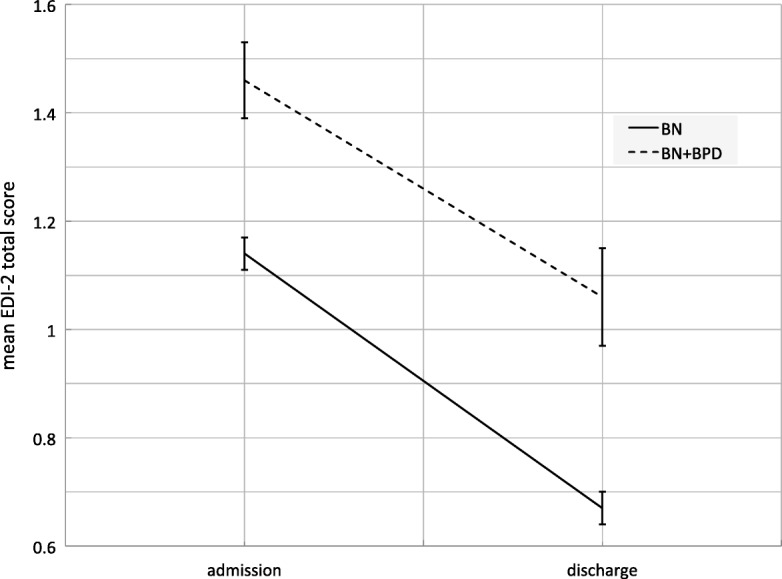
Main effect of treatment on EDI-2 total scores in inpatients with BN and BN + BPD. *Note*. Bars represent 95% confidence intervals. *EDI-2* Eating Disorder Inventory-2, *BN* bulimia nervosa, *BN + BPD* bulimia nervosa and Borderline Personality Disorder

**Fig. 2:**
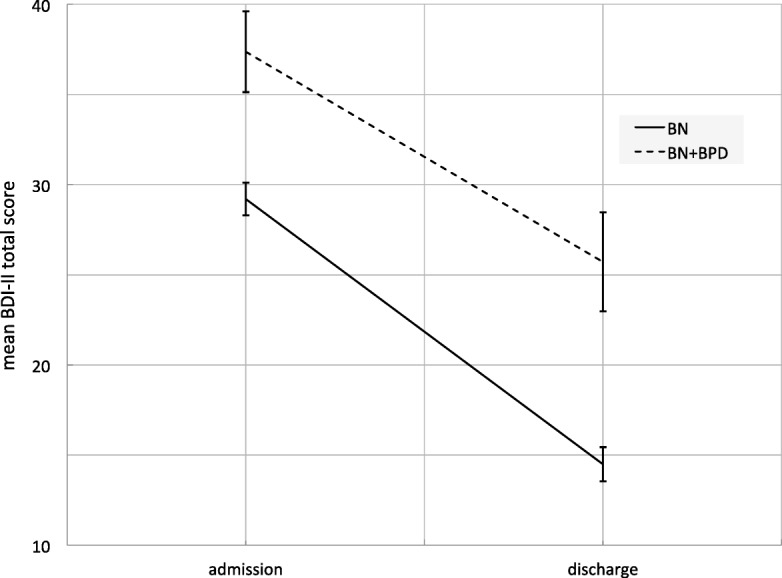
Interaction effect of treatment and diagnosis on BDI-II total scores in inpatients BN and BN + BPD. *Note*. Bars represent 95% confidence intervals. *BDI-II* Beck Depression Inventory-II, *BN* bulimia nervosa, *BN + BPD* bulimia nervosa and Borderline personality disorder

Even though patients with BN + BPD improved on all variables, their discharge scores often lay within the range of the admission scores of patients with BN. The respective 95% confidence intervals for BN + BPD discharge and BN admission scores overlapped for depressive symptoms, general psychopathology, as well as EDI-2’s total score and subscores for body dissatisfaction, ineffectiveness, perfectionism, interpersonal distrust, interoceptive awareness, maturity fears, asceticism, social insecurity. Figures 1 and 2 respectively exemplify a parallel slope for changes in EDI-2 total scores and an interaction in the treatment’s effect on depressive symptoms.

## Discussion

The aims of our study were to examine (1) the prevalence of comorbid BPD, (2) the specific characteristics of patients with comorbid BPD, and (3) differences in treatment course and outcome between comorbid and non-comorbid patients in a large sample of inpatients with BN. With regard to these aims we found that (1) 13% of the patients had a comorbid diagnosis of BPD; (2) more previous inpatient treatments, stronger weight gain during treatment, longer lengths of stay, fewer regular discharges, and lower rates of fitness to work at discharge in the comorbid group; as well as (3) improvements in eating disorder symptoms, depressive symptoms, general psychopathology, and psychosocial functioning during treatment in both groups, with the comorbid group showing less improvement and discharge scores in the range of the admission score of the BN-only group.

The prevalence of comorbid BPD in our sample is lower than what has been reported in meta-analyses, whose estimates range from one quarter [2] to one third [3, 4]. Martinussen and colleagues [2] argued that up to 50% of the variation in prevalence rates of BPD in eating disorders could be explained by the setting and the method of assessment, with lower rates resulting from outpatient samples and clinical interviews. This notion is in line with Zimmerman et al. (1999), who found marked differences in the prevalence of BPD when diagnosed in a structured (14.4%) vs. a clinical-unstructured (0.4%) way in outpatients. In our study with inpatients, the low rate might in part be explained by the fact that diagnoses were given after a standard clinical intake interview, which was not complemented with standardized personality disorder diagnostics. Another reason for the lower frequency of BPD in our sample might be a self-selection bias among patients with BPD in general. It is possible that these persons are aware of having a severe disorder and rather seek treatment in psychiatric settings. As a result, fewer of these patients would seek admission in our hospital, which has its focus on inpatient psychotherapy with adjuvant pharmacological treatment.

While patients with BN + BPD did not differ with regard to age, gender, admission weight, number of previous outpatient treatments, weekly weight gain, and discharge weight, we found a range of characteristics that were specific to patients with BN + BPD and usually indicated a worse status. Patients with BN + BPD reported around twice as many previous inpatient treatments, stayed 11 days longer in the hospital, and gained one kg more during treatment. Further, the comorbid patients were more often transferred to other hospitals due to somatic or psychiatric complications and prematurely discharged, resulting in a lower rate of regular discharges. Interestingly, more patients with BN than with BN + BPD discharged themselves against medical advice. At discharge, the comorbid patients were less often fit for work. These findings are novel and emphasize that patients with BN + BPD differ from patients with BN in variables that are relevant on personal, medical, and economic levels. Also, these results provide a complement from the health care perspective to the few cross-sectional studies that reported a worse psychiatric status and lower functioning for comorbid patients [12–14].

The results from our longitudinal analyses paint a mixed picture. One the one hand, both diagnostic groups, regardless of their diagnoses, improved during treatment in eating disorder symptoms, depressive symptoms, general psychopathology, and psychosocial functioning with moderate to large effect sizes. On the other hand, the comorbid group was more impaired on all measures at admission, showed less improvement in depressive symptoms, perfectionism, and asceticism during treatment, and was still markedly impaired at discharge. While bulimic symptoms changed for the better in both groups with a moderate effect size, impulse regulation and interpersonal problems, both shared symptoms of BN and BPD [12, 15] only improved with a small effect size. Importantly, in the majority of variables, the comorbid group showed discharge scores in the range of the admission scores of the BN-only group. For example, patients with BN were admitted with a mean BDI-II score corresponding to moderate depression [25, 26] and discharged with a score on the border between mild and minimal depression. Patients with BN + BPD had severe depression at admission and were discharged with moderate depression. In themselves already concerning, these findings have to be interpreted against the background of stronger weight gain, a longer stay in the current treatment, and more previous treatments. In general, our findings confirm and strongly extend the findings from previous studies that found more severe general psychopathology and poorer functioning in the comorbid patients [12, 13] as well as an improvement from very severe to severe eating disorder symptoms during specialized treatment [19]. They also contradict the proposed notion that eating disorder symptoms are not more pronounced in comorbid patients [15].

### Clinical implications

We found patients with BN + BPD to be severely impaired in both general and eating disorder psychopathology. Even after intense, specialized, and multimodal inpatient treatment, the patients still showed significant impairment and poor psychosocial function that in many cases prohibited taking over daily responsibilities. Especially core symptoms like impulse regulation, interpersonal distrust, and in the comorbid group even more depressive symptoms and perfectionism improved only to a small degree. The limited amenability of comorbid BN and BPD to treatment is emphasized by the results of two trials that were specifically designed to treat patients with both BPD and an eating disorder and reported high drop-out rates [20] and strong eating disorder symptoms at the end of the trial [19]. At best, BN + BPD seems to be treatable to a degree that patients can be transferred into outpatient treatment for further stabilization. To maximize outcomes, treatment should focus on transdiagnostic aspects of BN and BPD like impulse regulation, interpersonal problems, and depressive symptoms.

### Strengths and limitations

Strengths of our study include the large sample size and the longitudinal design, which was rarely employed in the study of comorbid BN and BPD. Further, our results reflect the current status in the German health care system. Limitations include that we were not able to conduct a follow-up after discharge and most measures rely on self-report, making them amenable to distortions. As a consequence of the routine care setting of our study, a substantial amount of missing data occurred, as patients might not return questionnaires, leave out items, which renders whole scales values non-interpretable, or were not administered questionnaires due to time constraints. Though the number of patients with complete data is low, the number of cases with complete data for the individual outcomes was sufficient. Also, only diagnoses from routine data were available and no standardized and structured interview was conducted.

## Conclusion

In conclusion, comorbid BN and BPD are associated with severe impairment, limited response to treatment, and a stronger use of the health care system. While these patients improve during specialized and intense inpatient treatment, substantial psychopathology residues and psychosocial functioning as well as the ability to work remain impaired. Treatment needs to be improved by focusing on shared aspects of the two seemingly distinct disorders.

## Additional file

Additional file 1: **Table S1.** Mean values for the repeated measures analyses of variance in Tables 2 and 3. The supplemental table displays the mean values for the dependent variables from the repeated measures analyses of variance. (PDF 55 kb)
