# Supplementary material for: Impact of comorbid borderline personality disorder on inpatient treatment for bulimia nervosa: analysis of routine data
Source: Borderline Personal Disord Emot Dysregul. 2019 Jan 16;6:1. doi: 10.1186/s40479-018-0098-4 (PMC6335811; doi:10.1186/s40479-018-0098-4)
Supplement: Supplementary file 1 — Table S1. Mean values for the repeated measures analyses of variance in Tables 2 and 3. The supplemental table displays the mean values for the dependent variables from the repeated measures analyses of variance. (PDF 55 kb) [file 40479_2018_98_MOESM1_ESM.pdf]

SUPPLEMENTAL TABLE 1. Mean values for the repeated measures analyses of variance in Tables 2 and 3.

| Variable | Time | BN, *M* (*SD*) | BN+BPD, *M* (*SD*) |
|---|---|---|---|
| EDI-2 total score | Admission | 1.14 (0.45) | 1.46 (0.39) |
| | Discharge | 0.67 (0.44) | 1.06 (0.50) |
| EDI-2 drive for thinness | Admission | 1.91 (0.88) | 2.32 (0.68) |
| | Discharge | 1.03 (0.90) | 1.60 (0.97) |
| EDI-2 bulimia | Admission | 1.41 (0.80) | 1.54 (0.76) |
| | Discharge | 0.27 (0.50) | 0.42 (0.55) |
| EDI-2 body dissatisfaction | Admission | 1.98 (0.89) | 2.41 (0.64) |
| | Discharge | 1.45 (0.96) | 2.04 (0.90) |
| EDI-2 ineffectiveness | Admission | 1.09 (0.68) | 1.61 (0.62) |
| | Discharge | 0.60 (0.60) | 1.17 (0.73) |
| EDI-2 perfectionism | Admission | 1.06 (0.73) | 1.16 (0.81) |
| | Discharge | 0.77 (0.63) | 1.03 (0.77) |
| EDI-2 interpersonal distrust | Admission | 0.84 (0.59) | 1.19 (0.63) |
| | Discharge | 0.55 (0.52) | 0.86 (0.63) |
| EDI-2 interoceptive awareness | Admission | 1.16 (0.68) | 1.55 (0.65) |
| | Discharge | 0.61 (0.61) | 1.10 (0.71) |
| EDI-2 maturity fears | Admission | 0.84 (0.64) | 0.93 (0.67) |
| | Discharge | 0.62 (0.53) | 0.74 (0.62) |
| EDI-2 sexual asceticism | Admission | 0.89 (0.52) | 1.07 (0.51) |
| | Discharge | 0.52 (0.47) | 0.82 (0.58) |
| EDI-2 impulse regulation | Admission | 0.54 (0.46) | 1.00 (0.56) |
| | Discharge | 0.34 (0.42) | 0.77 (0.59) |
| EDI-2 social insecurity | Admission | 1.00 (0.56) | 1.38 (0.52) |
| | Discharge | 0.65 (0.52) | 1.09 (0.56) |
| BDI-II | Admission | 29.20 (11.19) | 37.37 (10.54) |
| | Discharge | 14.49 (11.74) | 25.71 (12.85) |
| GSI | Admission | 1.34 (0.75) | 1.80 (0.74) |
| | Discharge | 0.82 (0.64) | 1.39 (0.77) |
| GAF | Admission | 46.82 (9.96) | 42.93 (7.84) |
| | Discharge | 61.34 (12.64) | 55.88 (9.21) |
| BMI | Admission | 22.25 (4.33) | 22.78 (4.46) |
| | Discharge | 22.65 (4.01) | 23.56 (4.12) |

*Note*. *M* = mean, *SD* = standard deviation, EDI-2 = Eating Disorder Inventory 2, BDI-II = Beck Depression Inventory II, GSI = Global Severity Index, GAF = Global Assessment of Functioning, BMI = body mass index.
